# A cornucopia of research resources for the fourth rodent malaria parasite species

**DOI:** 10.1186/s12915-021-01019-y

**Published:** 2021-04-23

**Authors:** Jane M. Carlton

**Affiliations:** Center for Genomics & Systems Biology, Department of Biology, New York University, 12 Waverly Place, New York, NY 10003 USA

## Abstract

The study of human malaria caused by species of *Plasmodium* has undoubtedly been enriched by the use of model systems, such as the rodent malaria parasites originally isolated from African thicket rats. A significant gap in the arsenal of resources of the species that make up the rodent malaria parasites has been the lack of any such tools for the fourth of the species, *Plasmodium vinckei*. This has recently been rectified by Abhinay Ramaprasad and colleagues, whose pivotal paper published in BMC Biology describes a cornucopia of new *P. vinckei* ‘omics datasets, mosquito transmission experiments, transfection protocols, and virulence phenotypes, to propel this species firmly into the twenty-first century.

## The “RMPs”

Since their discovery in wild thicket rats and mosquitoes in the forests of sub-Saharan Africa in the middle of the last century [1], rodent malaria parasites known as “RMPs” have provided an informative model system for furthering our knowledge of human malaria parasites (HMPs) such as *Plasmodium falciparum* and *Plasmodium vivax*, as well as insights into a lineage that is fascinating in its own right. Four species were originally identified: *Plasmodium berghei* and *Plasmodium yoelii* (which both invade reticulocytes) form one clade, while *Plasmodium chabaudi* and *Plasmodium vinckei* (which preferentially invade mature red blood cells) form another. All have been adapted to growth in laboratory mice and rats (and several transmitted through laboratory colonies of *Anopheles*), but only the first three species have been developed as model systems for the study of various malaria phenomena (reviewed in [2]). For example, *P. yoelii* has been used extensively to study the challenging pre-erythrocytic stages of the malaria life cycle, resulting in an extensive toolkit of resources including promising methods for the study of the dormant “relapse” liver stage of *P. vivax* [3]. *P. berghei* on the other hand has been developed as a force for reverse genetics; as an example, transgenic knockout lines have been developed and phenotyped for almost half of the genes in the genome [4], with DNA vectors and other tools available through the online resource *Plasmo*GEM (https://plasmogem.sanger.ac.uk). Finally, the synchronous species *P. chabaudi* has proven valuable for experimental evolution studies such as selecting antimalarial drug resistant mutants using increasing drug selective pressure in vivo and undertaking genetic crossing experiments to identify loci associated with their phenotypes [5]. Efficient transfection techniques have been developed in all three species, as well as high quality reference genomes.

Quite why these three species were promoted, developed and primed into the models used today, but *P. vinckei* was not, is unclear. In fact, *P. vinckei* is the most geographically widespread of the four RMPs, found in all five of the sub-Saharan countries in Africa (Cameroon, Central African Republic, Congo, Democratic Republic of the Congo, Nigeria) from which RMPs have been isolated. At least four subspecies have been identified (*Plasmodium vinckei vinckei*, *Plasmodium vinckei petteri*, *Plasmodium vinckei lentum*, and *Plasmodium vinckei brucechwatti*), and at least 18 genetically different isolates (according to isoenzyme analysis) exist. *Anopheles dureni millecampsi* has also been identified as its mosquito vector [1]. This is in sharp contrast to the paucity of available genetically diverse *P. berghei* isolates; for a species that is at the forefront of reverse genetics, *P. berghei* thus has its limitations. Isolates, clones, and lines of *P. vinckei* and the other RMPs are diligently maintained by the European Malaria Reagent Repository (http://www.malariaresearch.eu/content/rodent-malaria-parasites).

## A cornucopia of *P. vinckei* resources

The paper by Abhinay Ramaprasad and colleagues [6] redresses this imbalance. First, a thorough analysis of the virulence phenotype of eight new clones of various *P. vinckei* subspecies identified extensive diversity, setting the stage for subsequent studies of this important trait. Subsequently, next generation sequencing was used to generate high-quality reference genomes of each of the four named subspecies and a fifth newly named *Plasmodium vinckei baforti,* and the genotypic diversity of the species—only hinted at by previous isoenzyme analyses—explored. A comparative genomics analysis comprising deep sequencing of seven isolates of *P. chabaudi* and *P. yoelii* was also achieved, resolving evolutionary relationships among the RMPs and identifying positively selected genes such as several involved in mosquito transmission. Next, the team integrated transcriptomic data with the high-quality reference genome annotations to describe patterns of evolution of the many multigene families both within the subspecies of *P. vinckei* and between *P. vinckei* and all RMPs. Interestingly, both the levels of gene expression and their life cycle stage specificities were generally conserved across the RMPs, signifying that orthologs in structurally distinct clades may have conserved functions across the different species. One subspecies, *P. v. vinckei*, was found to have a much reduced repertoire of multigene families. Genetic crossing experiments with different *P. vinckei* subspecies were successful (albeit with varying levels of recombinant progeny produced) and transfection of one *P. v. vinckei* clone produced a green fluorescent protein–firefly luciferase transfectant that was stable through asexual and mosquito stages.

What a rich set of resources! Whereas the development of a model system once took decades, with the aid of next generation sequencing (especially so-called long read technology) and enhanced molecular biology techniques, Ramaprasad et al. [6] have fast-tracked the establishment of *P. vinckei* as a useful additional experimental model for malaria.

## How can a mouse model be so controversial?

**Fig. 1.**
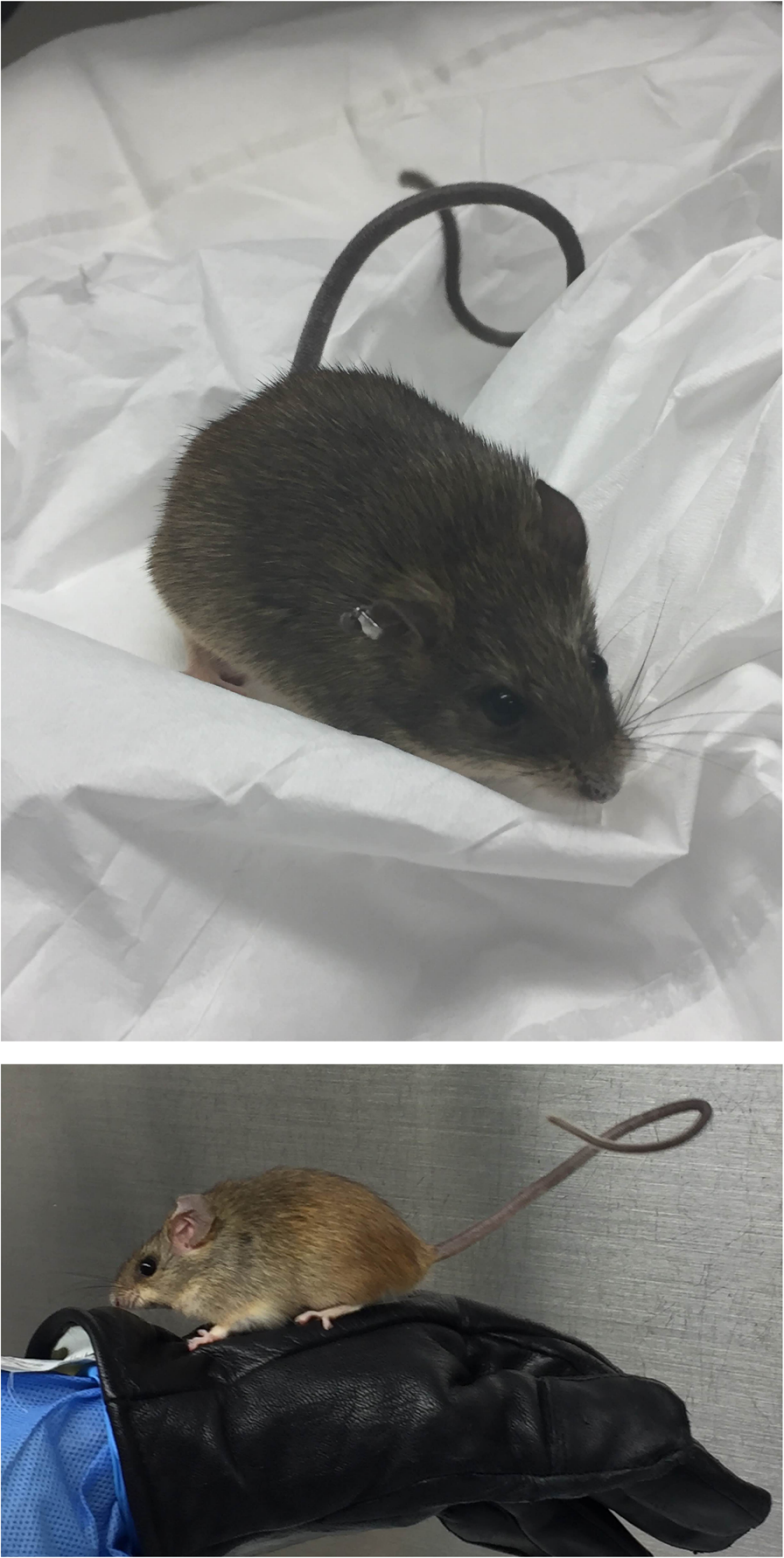
Photos of a thicket rat *Grammomys surdaster* from an active colony. Unlike the short tails of domestic lab mice which can be used to pick them up, thicket rats have very long tails which provide traction and enable the rodents to escape capture by twirling and twisting around. Photos courtesy of Dr. Patrick Duffy and Ms. Lynn E. Lambert, National Institutes of Health, National Institute of Allergy and Infectious Diseases, Laboratory of Malaria Immunology and Vaccinology, Rockville, MD, USA

The use of RMPs as models for studying aspects of human malaria has not been without controversy. In particular, the use of the *P. berghei* model for the study of pathogenesis and cerebral malaria has been criticized for its relevance [7, 8], with a flurry of papers proposing an alternative or more nuanced view in response (see for example [8]). A proposal put forward recently is that it is the very *differences* between the RMPs and HMPs and the interactions with their mammalian hosts that should be noted and exploited [9]. Other studies have proposed reverting to use of the known natural host of several of the RMPs, the African thicket rat *Grammomys* (Fig. 1), for experimental studies on vaccines or adjunct therapies, because it represents a more rigorous model with a range of malaria infection outcomes including chronic parasitemia, clearance, or rapid, lethal infection, compared to infections of RMPs in laboratory mice [10]. The thicket rat, whose typical habitat is small shrubs and trees in tropical forest, is a nocturnal rodent that lives in nests 2–3 m above the forest ground and has a highly specialized activity cycle for forest conditions, lying immobile on branches for long periods of time, rendering the rodent as an easy evening snack or early morning breakfast for its mosquito vector.

## The utility of RMPs in malaria research: the future

What does the availability of multiple reference genomes, phenotypic data, transmission, and transfection protocols, for a fourth species of RMP mean? There is no doubt that over the past 60 years, rodent malaria parasite species have provided an informative model system for the study of malaria parasite biology, whether that can be directly extrapolated to human malaria parasites species or not. The order Haemosporida consists of more than 500 described malaria parasite species from at least 15 genera that infect mammals, birds, and reptiles throughout the world and are transmitted by several clades of blood-feeding insects [11]. An additional set of genetic and genomic resources that fills in the gap of the fourth described RMP, especially one with such a rich diversity of subspecies and isolates with a known natural mammalian host and vector, can only aid in furthering our understanding of this fascinating group of species.

## Data Availability

Not applicable
